# MnO_2_ Nanoparticles Decorated PEDOT:PSS for High Performance Stretchable and Transparent Supercapacitors

**DOI:** 10.3390/nano14131080

**Published:** 2024-06-24

**Authors:** Guiming Liu, Zhao Huang, Jiujie Xu, Tiesong Lin, Bowen Zhang, Peng He

**Affiliations:** 1 State Key Laboratory of Precision Welding & Joining of Materials and Structures, Harbin Institute of Technology, Harbin 150001, China; liu.guiming@outlook.com (G.L.); huangzhaohit@outlook.com (Z.H.); 20b909121@stu.hit.edu.cn (J.X.); hitjoining@hit.edu.cn (T.L.); 2 School of Electrical Engineering, Tiangong University, Tianjin 300350, China

**Keywords:** supercapacitors, MnO_2_, PEDOT:PSS, nanoparticles, stretchable, transparent

## Abstract

With the swift advancement of wearable electronics and artificial intelligence, the integration of electronic devices with the human body has advanced significantly, leading to enhanced real-time health monitoring and remote disease diagnosis. Despite progress in developing stretchable materials with skin-like mechanical properties, there remains a need for materials that also exhibit high optical transparency. Supercapacitors, as promising energy storage devices, offer advantages such as portability, long cycle life, and rapid charge/discharge rates, but achieving high capacity, stretchability, and transparency simultaneously remains challenging. This study combines the stretchable, transparent polymer PEDOT:PSS with MnO_2_ nanoparticles to develop high-performance, stretchable, and transparent supercapacitors. PEDOT:PSS films were deposited on a PDMS substrate using a spin-coating method, followed by electrochemical deposition of MnO_2_ nanoparticles. This method ensured that the nanosized MnO_2_ particles were uniformly distributed, maintaining the transparency and stretchability of PEDOT:PSS. The resulting PEDOT:PSS/MnO_2_ nanoparticle electrodes were gathered into a symmetric device using a LiCl/PVA gel electrolyte, achieving an areal capacitance of 1.14 mF cm^−2^ at 71.2% transparency and maintaining 89.92% capacitance after 5000 cycles of 20% strain. This work presents a scalable and economical technique to manufacturing supercapacitors that combine high capacity, transparency, and mechanical stretchability, suggesting potential applications in wearable electronics.

## 1. Introduction

With the swift advancement of biology, electronics, and artificial intelligence, wearable electronic devices are increasingly being integrated onto the human body to collect various physiological and biochemical information for health monitoring and disease diagnosis [1,2,3]. Although researchers have developed stretchable materials with skin-like mechanical properties to improve comfort and the quality of bio-signals, their optical properties are in the early stages of consideration [4,5,6]. Optical transparency is crucial in wearable electronic devices because it makes the devices less noticeable and more aesthetically pleasing, thereby enhancing users’ willingness to wear them. Additionally, transparent devices can maintain visual information at the contact areas with the body, improving understanding of health conditions. For example, when users wear transparent wearable electronic devices for surgical treatment, the visual information of the injury site can be observed, providing the most intuitive and efficient data for analyzing the healing process [7]. Therefore, there is an urgent need to develop stretchable and transparent components for wearable devices, including electrodes, sensors, energy systems and displays [8,9,10,11,12,13].

Among energy devices, supercapacitors are considered as one of the most viable solutions for energy storage. Fuel cells have extremely high energy density but suffer from kinetic sluggishness (low power density) and expensive metal catalysts [14,15]. Batteries have short lifespans and slow charging rates due to limitations, such as solid-state diffusion rates, phase transformations, and volume changes during charge and discharge cycles [16,17,18]. Additionally, their energy density drops sharply with size, limiting their application in wearable and small-sized electronics [19]. In contrast, supercapacitors exhibit characteristics such as portability, long lifespan, rapid charge/discharge rates, high energy density, low operational costs, and excellent safety, making them widely used in wearable and portable devices [20,21,22,23]. However, fabricating supercapacitors with high capacitance, stretchability, and transmittance remains a substantial challenge.

Poly(3,4-ethylenedioxythiophene):poly(styrene sulfonate) (PEDOT:PSS), as one of the most prevalent conductive polymers, finds extensive application in the fabrication of photovoltaics, panels, transistors, and various sensors due to its advantages such as transparency in the visible light range (over 90%), tunable conductivity (1 to 4000 S cm^−1^) and work function, stretchability (over 100% strain once doped), and solution processability [24,25,26,27]. Additionally, PEDOT:PSS has comparatively high capacity and electrochemical stability, rendering it ideal for fabricating stretchable and transparent supercapacitors [28]. PEDOT:PSS was directly used as the active material for supercapacitors during early studies. In 2015 and 2016, Coleman et al. and Huang et al. used spray coating and spin coating methods, respectively, to fabricate PEDOT:PSS films on polyethylene terephthalate (PET) substrates, forming supercapacitor electrodes. The latter further used gel electrolyte to assemble two electrodes into a solid-state supercapacitor. Subsequently, to meet the demand for higher capacitance in practical applications, researchers focused on developing supercapacitors that mix PEDOT:PSS with high specific capacitance materials. Transition metal oxides have garnered extensive attention as pseudo-capacitive materials due to their high specific capacity [29,30,31]. Transition metals have multiple oxidation states, which allow for fast reversible Faradaic redox reactions during electrode charge and discharge processes, thus storing and releasing electrons [32,33]. Zhang et al. dispersed RuO_2_ nanoparticles (NPs) in a PEDOT:PSS solution and deposited it onto a PET substrate by spray-coating method to fabricate a flexible and transparent supercapacitor [34]. The PEDOT:PSS/RuO_2_ NPs symmetric solid-state device displayed 80% transmittance and a specific capacitance of 0.84 mF cm^−2^, which is 73.7% higher than that of a PEDOT:PSS based supercapacitor with similar transmittance. Huang et al. coated silver nanowires, Na_x_WO_3_ nanowires, and PEDOT:PSS on a PET substrate to serve as one electrode and used PEDOT:PSS/PET film as another electrode, with gel electrolyte, to assemble a flexible transparent supercapacitor [35]. This asymmetric device presented an areal capacitance of 1.11 mF cm^−2^, despite that its transparency was only 55%. However, the transition metal elements used in these studies have low abundance in the Earth’s crust, resulting in high costs. Additionally, the materials were prepared using hydro-thermal methods, which are complex and further increase costs. More importantly, these fabricated supercapacitors only possess a flexibility which did not fully utilize the stretchability of PEDOT:PSS.

Although MnO_2_ has been widely deposited on various current collectors to prepare a supercapacitor electrode, the continuous distribution of MnO_2_ renders these electrodes without transparent or stretchable properties. This study is the first to achieve dispersed deposition of MnO_2_ particles with diameters less than 200 nm onto a transparent and stretchable PEDOT:PSS film. This unique structure endows the electrode with excellent capacitance while remaining both transparent and stretchable. On the one hand, the nanosized MnO_2_ particles have high surface activation energy and specific surface area, fully exploiting the high specific capacitance characteristics of MnO_2_. On the other hand, the dispersed distribution of MnO_2_ nanoparticles preserves the intrinsic transparent and stretchable properties of PEDOT:PSS. Furthermore, compared to other methods that use hydro-thermal processes to prepare rare metal compounds for enhancing PEDOT:PSS performance [34,35], the electrochemical deposition of MnO_2_ used in this study offers advantages of low cost and high efficiency. The Mn element is earth-abundant and cost-effective, while the solution processability of the preparation process makes this electrode material suitable for large-scale manufacturing, thereby further decreasing costs. We assembled the PEDOT:PSS/MnO_2_ NP electrodes into a symmetric supercapacitor using a gel electrolyte, achieving an areal capacitance of 1.09 mF cm^−2^ with 71.1% transparency, which is 172% higher than that of PEDOT:PSS supercapacitors with similar transmittance. Moreover, this device maintained 90.44% of its initial capacity even after 5000 cycles with 20% strain stretching, indicating good stretchable stability. As a prototype device, the proposed PEDOT:PSS/MnO_2_ NP supercapacitor successfully combines high capacity, high transparency and excellent mechanical stretchability, suggesting a promising application towards wearable electronics.

## 2. Experimental

**Figure 1:**
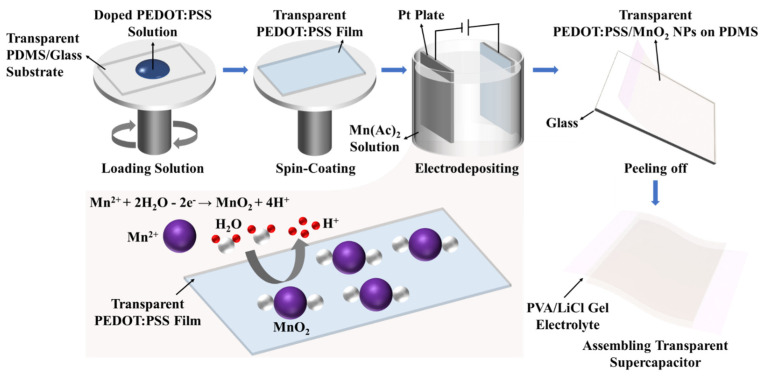
Manufacturing steps of PEDOT:PSS/MnO_2_ NPs stretchable and transparent supercapacitor.

Figure 1 shows the fabrication process for PEDOT:PSS/MnO_2_ NP stretchable and transparent supercapacitors.

### 2.1. Materials

Unless otherwise noted, materials were obtained from Aladdin (Shanghai, China).

### 2.2. Preparation of PEDOT:PSS Electrodes

Firstly, PDMS obtained from Dow Corning (Midland, MI, USA) was mixed thoroughly at a ratio of 10:1 (base/crosslinker). After the bubbles disappeared, this was deposited onto a glass substrate and heated in a 60 °C oven for 3 h. The resulting PDMS/glass was cut to the desired size for subsequent use. 7.8 g of the original PEDOT:PSS solution (Clevios PH1000, obtained from Heraeus, Hanau, Germany) was blended with 0.2 g Triton X-100, 0.5 g ethylene glycol, and 1.5 g Capstone FS-3100 (obtained from DuPont, Wilmington, DE, USA) to enhance the conductivity and stretchability of PEDOT:PSS [36,37,38]. The doped PEDOT:PSS solution was deposited onto the surface by spin-coating method at different speeds, then dried in a 60 °C oven for 10 min. Silver paste was applied to the ends and, after curing, copper conductive tape was attached. These areas were then sealed with PDMS. 

### 2.3. Preparation of PEDOT:PSS/MnO_2_ NP Electrodes

The MnO_2_ NPs were deposited onto the PEDOT:PSS electrode by using a two-electrode configuration, consisting of the PEDOT:PSS film as the working electrode, and platinum foil as the counter electrode at room temperature. The electrolyte solution contained 100 mM manganese acetate tetrahydrate (Mn(AC)_2_) and MnO_2_ NPs was grown by passing a constant area current of 0.33 mA cm^−2^ for 10 to 60 s. Subsequently, the prepared PEDOT:PSS/MnO_2_ NP electrodes were soaked in distilled water to eliminate residual electrolyte. The thickness of the electrode is 0.3 mm.

### 2.4. Preparation of PEDOT:PSS and PEDOT:PSS/MnO_2_ NP Devices

5.0 g poly(vinyl alcohol) (PVA 1788) and 5.0 g lithium chloride (LiCl) were added to 50.0 g deionized water, stirred at room temperature until the solution clarified, and LiCl/PVA gel electrolyte was obtained. It was drop-coated onto the electrodes and placed at room temperature for 5 h. After that, PEDOT:PSS or PEDOT:PSS/MnO_2_ NPs with PDMS were peeled off from the glass substrate, and two identical electrodes were pressed together to form symmetric solid-state supercapacitors. The thickness of the assembled device is 1.2 mm.

### 2.5. Electrodes and Supercapacitors‘ Characterization

SEM pictures were acquired using a Hitachi SU5000 electron microscope (Tokyo, Japan) operating with an accelerating voltage of 2 kV. TEM pictures were captured using a Talos F200X transmission electron microscope (Waltham, MA, USA) operating with an accelerating voltage of 300 kV and coupled with an energy-dispersive X-ray spectroscopy (EDS) detector (Oxford, UK). XPS analysis was performed on a Thermo Scientific Nexsa spectrometer (Waltham, MA, USA). XPS spectra were adjusted to a peak C 1s binding energy of 284.8 eV. AFM images were obtained by a diInnova SPM instrument (Billerica, MA, USA) with silicon probes.

Optical transmittance measurements were carried out using a UV–VIS spectrophotometer (Shimadzu UV-2600, Kyoto, Japan), with air serving as the reference. Electrochemical performance was evaluated using an electrochemical workstation (CHI660, Shanghai, China). The electrochemical performance of the electrode was evaluated by a three-electrode setup, using an Ag/AgCl reference electrode, a platinum plate counter electrode, a PEDOT:PSS or PEDOT:PSS/MnO_2_ NPs working electrode, and 1 M LiCl aqueous electrolyte. The electrochemical properties of the devices were tested using a two-electrode system. The effective area of both the electrode and the device is 3 cm^2^ (1.5 cm × 2.0 cm). The areal capacitance was calculated from the CV curves. In order to characterize the stretchability of the supercapacitor, it was fixed onto a homemade motorized stretching device and subjected to various strains with a speed of 1 mm s^−1^ along the 1.5 cm edge.

## 3. Results and Discussion

### 3.1. Analysis of PEDOT:PSS/MnO_2_ NP Films

**Figure 2:**
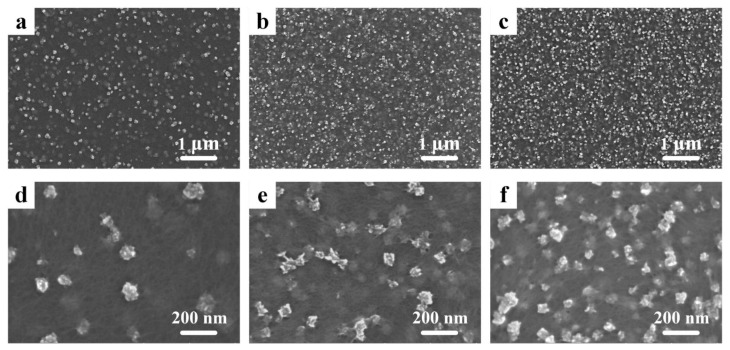
SEM pictures of the PEDOT:PSS/MnO_2_ NP films with different deposition times. (**a**,**d**) 10 s. (**b**,**e**) 30 s. (**c**,**f**) 60 s.

Scanning electron microscopy (SEM) pictures of PEDOT:PSS/MnO_2_ NP films with different electrochemical deposition times can be seen in Figure 2. Rough-surfaced particles with diameters less than 200 nm are randomly distributed on the surface of PEDOT:PSS. These zero-dimensional nanostructures have a large specific surface area, which allows for ample contact with the electrolyte during charge and discharge processes, enhancing energy storage performance [32]. It is worth noting that, as the deposition time increases, the number of nanoparticles gradually increases, further enlarging the specific surface area of the film, while the diameter of the nanoparticles does not increase (Table S1). This effectively avoids the problem of rapid degradation in capacitance performance due to the increased resistance of transition metal oxides as particle size increases [33].

**Figure 3:**
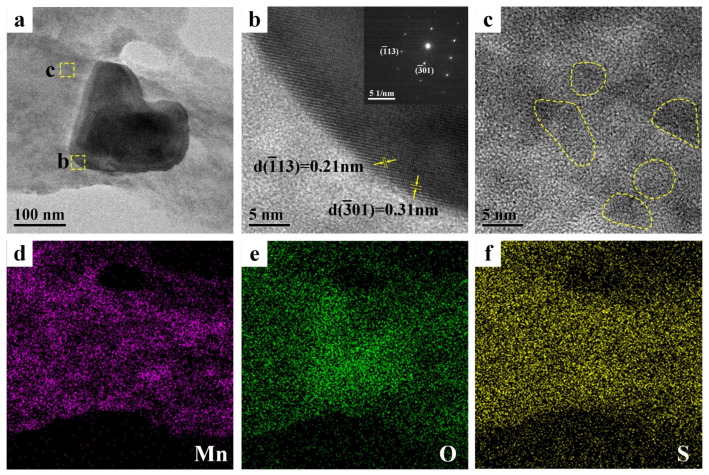
TEM pictures and EDS mappings of the PEDOT:PSS/MnO_2_ NP films. (**a**) TEM picture. (**b**) HRTEM image from the dashed box area in (**a**) and corresponding electron diffraction pattern. (**c**) HRTEM image from the dashed box area in (**a**). EDS mappings of (**a**): (**d**) Mn, (**e**) O and (**f**) S.

The morphology and phase composition of PEDOT:PSS/MnO_2_ NP film were further analyzed by transmission electron microscope (TEM), as shown in Figure 3. There is a particle with a diameter of approximately 150 nm on the PEDOT:PSS film (Figure 3a). HRTEM and electron diffraction can determine that the structure of this particle is α-MnO_2_ (Figure 3b). TEM analysis of other area in Figure 3a shows that MnO_2_ particles with diameters less than 10 nm are also distributed on the PEDOT:PSS film (marked with circles in Figure 3c). These extremely small MnO_2_ NPs are beneficial for enhancing the energy storage capacity of the electrode. EDS analysis of the PEDOT:PSS/MnO_2_ NP films (Figure 3d–f) also shows that Mn elements are almost uniformly distributed on the surface of PEDOT:PSS.

**Figure 4:**
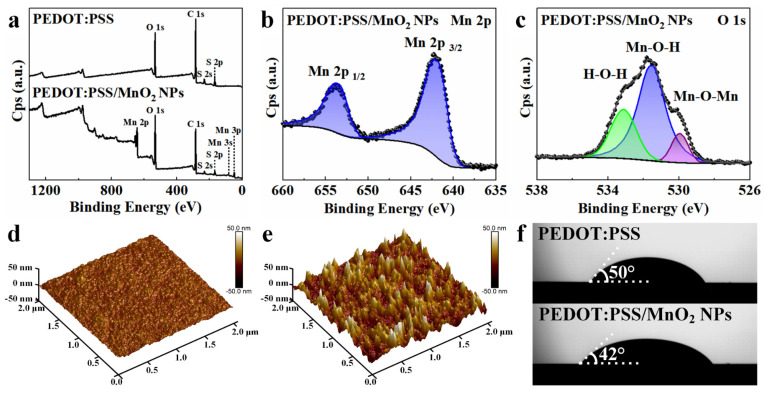
Surface analysis of PEDOT:PSS and PEDOT:PSS/MnO_2_ NP films. (**a**) XPS full spectra of PEDOT:PSS and PEDOT:PSS/MnO_2_ NP films. (**b**) XPS Mn 2p and (**c**) O 1s spectra of PEDOT:PSS/MnO_2_ NP film. AFM pictures of (**d**) PEDOT:PSS film and (**e**) PEDOT:PSS/MnO_2_ NP film. (**f**) Photographs of LiCl/PVA droplets on PEDOT:PSS and PEDOT:PSS/MnO_2_ NP films.

XPS spectra were used to access the oxidation state of the deposited MnO_2_ NPs. The XPS full spectrum of the PEDOT:PSS film (Figure 4a) contains peaks of O, S, and C elements, while the XPS full spectrum of the PEDOT:PSS/MnO_2_ NP film additionally contains a peak of Mn, indicating that MnO_2_ NPs are deposited on the surface of PEDOT:PSS. The Mn 2p spectrum (Figure 4b) shows double peaks for Mn 2p _3/2_ and Mn 2p _1/2_ with binding energies at 642.3 eV and 654.0 eV, respectively, with an energy separation of 11.7 eV. This aligns with previously reported data for MnO_2_, suggesting that the oxidation state of Mn is +4 [39]. The O 1s XPS curve (Figure 4c) can be analyzed in detail into three peaks at 533.1 eV, 531.7 eV, and 530.0 eV, aligning with H-O-H in residual water, M-O-H in hydroxides, and Mn-O-Mn in oxides, respectively [40].

The morphology of the PEDOT:PSS film before and after deposition of MnO_2_ NPs was analyzed by Atomic Force Microscopy (AFM). After deposition, the structure of the PEDOT:PSS film changed significantly, with a marked increase in roughness, as shown in Figure 4d,e. The arithmetic average roughness (Ra) and root mean square roughness (Rq) increased from 1.51 nm and 1.94 nm before deposition to 7.96 nm and 10.3 nm after deposition, respectively. This sharp increase in surface area is beneficial for the exchange of electrons and ions between the electrode material and the electrolyte during charge-discharge processes, thereby enhancing the energy storage performance of the electrode. The contact angle of the LiCl/PVA solution on the PEDOT:PSS film is 50°, which decreases to 42° on the PEDOT:PSS/MnO_2_ NP film. This indicates that the deposition of MnO_2_ NPs improved the hydrophilicity of the PEDOT:PSS film, which is highly desirable for the rapid access of ions to the electrode–electrolyte interface during electrochemical reaction.

### 3.2. Characterization of PEDOT:PSS/MnO_2_ NP Electrodes

**Figure 5:**
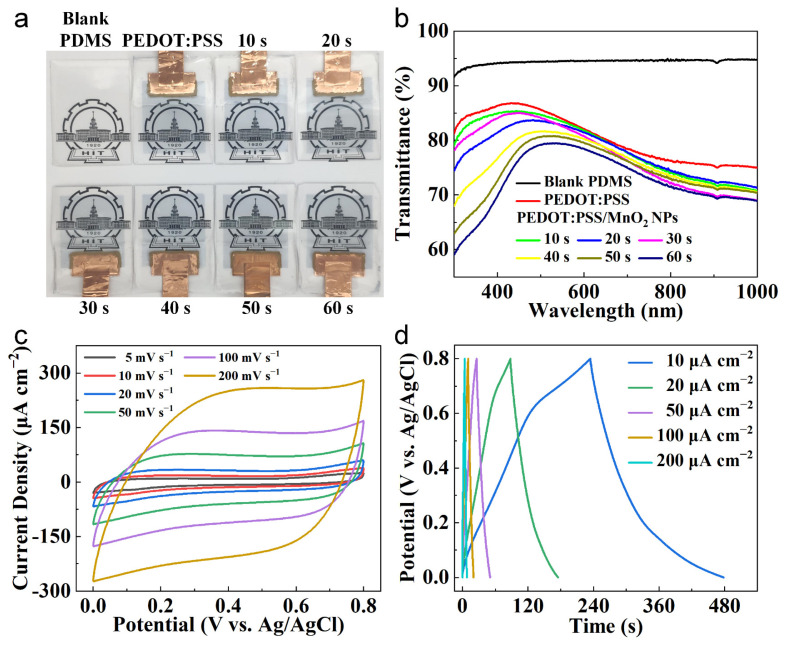
Transparency and electrochemical characteristics of the PEDOT:PSS/MnO_2_ NP Electrodes. (**a**) Digital photos and (**b**) Transmittance spectra (using air as the reference) of blank PDMS, PEDOT:PSS electrode and PEDOT:PSS/MnO_2_ NP electrodes with various deposition time. (**c**) CV curves and (**d**) GCD curves of the PEDOT:PSS/MnO_2_ NP electrode with deposition time 40 s.

Figure 5a shows the photographs of the electrodes made with PEDOT:PSS/MnO_2_ NPs. With the increase in MnO_2_ NPs electrochemical deposition time, the color of the electrode gradually deepens, but it still maintains relatively high transparency. The optical transmittance tests were conducted on blank PDMS, PEDOT:PSS electrodes, and PEDOT:PSS/MnO_2_ NP electrodes. As shown in Figure 5b, the transparency of blank PDMS (at 550 nm, same below) is 94.53%, PEDOT:PSS electrode is 83.95%, and PEDOT:PSS/MnO_2_ electrode ranges from 83.45% to 79.36%.

The electrochemical property of the PEDOT:PSS/MnO_2_ NP electrodes was measured at room temperature using a three-electrode configuration, including an Ag/AgCl reference electrode, a platinum foil counter electrode, a working electrode, and 1 M LiCl aqueous as the electrolyte. Taking the deposition time of 40 s as an example, the CV curves of the PEDOT:PSS/MnO_2_ electrode with scan rates of 5–200 mV s^−1^ are shown in Figure 5c. The nearly rectangular and symmetrical CV curves indicate that the electrode has good charge storage characteristics and reversible redox transitions, and this shape remains consistent to 200 mV s^−1^. This good capacitive property was also confirmed by GCD test. The almost triangular-shaped curves at area current of 5–200 μA cm^−2^ (Figure 5d) indicate rapid ion transport between the electrode and the electrolyte.

**Figure 6:**
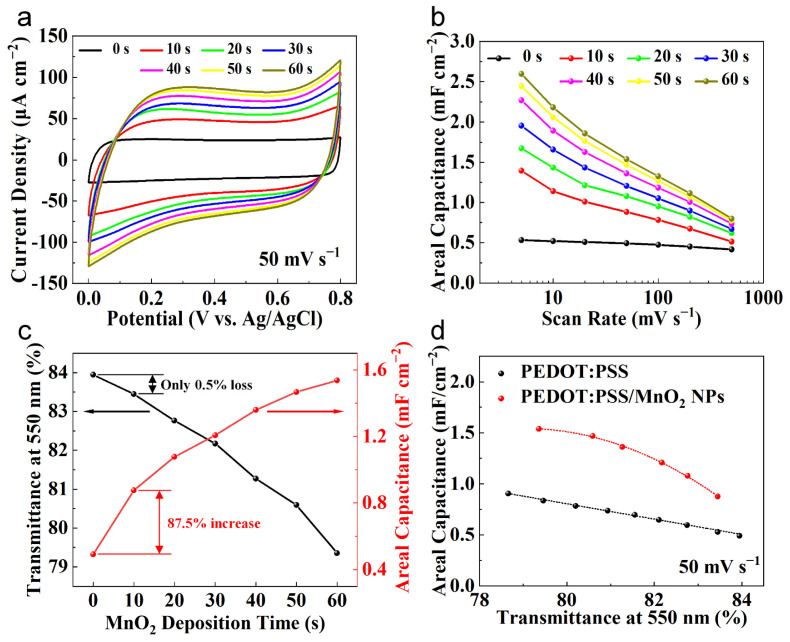
Transparency and electrochemical properties of PEDOT:PSS and PEDOT:PSS/MnO_2_ NP electrodes. (**a**) CV curves of PEDOT:PSS electrode and PEDOT:PSS/MnO_2_ NP electrodes with different deposition time. (**b**) The areal capacitance of different electrodes at different scan rate, which were calculated by the CV curves. (**c**) Transmittance and areal capacitance versus PEDOT:PSS/MnO_2_ NPs deposition time relationships. (**d**) Areal capacitance versus electrode transparency with a sweep rate of 50 mV s^−1^.

The relationship between the electrochemical performance and transparency of PEDOT:PSS/MnO_2_ NP electrodes with the deposition time was systematically studied. Figure 6a shows the CV curves of various electrodes at a sweep rate of 50 mV s^−1^. The CV curve of the PEDOT:PSS electrode presents a nearly square shape, demonstrating a good capacitive response. With the deposition time expands, the area enclosed by the CV curves gradually enhances, suggesting the pseudo-capacitance provided by the deposited MnO_2_ NPs. Its energy storage mechanism is charge transferred through the Faradaic redox reaction between Mn^4+^ and Mn^3+^ in the electrolyte [29,41]:

MnO_2_ + Li^+^ + e^−^ ↔ MnOOLi (1)

Figure 6b shows the areal capacitance of the electrode at different charge/discharge speeds, obtained from the CV curves. The areal capacitance of the electrode gradually enhances with the deposition time of MnO_2_ NPs. When the deposition time is 60 s, the areal capacitance of the PEDOT:PSS/MnO_2_ NP Electrode at 50 mV s^−1^ is 2.60 mF cm^−2^, which is 390% higher than that of the PEDOT:PSS electrode. Even at 500 mV s^−1^, the areal capacitance of the PEDOT:PSS/MnO_2_ NP electrode remains at 0.80 mF cm^−2^, which is 90% higher than that of the PEDOT:PSS electrode. The difference in performance improvement at different scan rates is primarily owing to the greater impact of electrode resistance on capacitance at high sweep rate.

The relationship curves between the areal capacitance and transparency of the electrodes with the deposition time of MnO_2_ NPs are plotted in Figure 6c. As the deposition time expands, the transparency gradually declines while the areal capacitance gradually increases. In contrast to the PEDOT:PSS electrode, when the deposition time is 10 s, the Transmittance of the PEDOT:PSS/MnO_2_ NP electrode decreases by only 0.5% (from 84.0% to 83.5%), while the areal capacitance increases significantly by 87.5% (from 0.49 mF cm^−2^ to 0.88 mF cm^−2^). The dispersed distribution mode causes the nanoparticles to have minimal impact on the transparency of the electrode. Additionally, the nanoscale MnO_2_ particles possess an ultra-high specific surface area, which contributes to fully utilize the high specific capacitance characteristics of MnO_2_. Nevertheless, as the deposition time expands, the transparency of the electrode decreases more rapidly, while the increase in areal capacitance slows down, which is caused by the increased resistance due to the larger quantity of MnO_2_ NPs.

The optical performance and electrochemical property of PEDOT:PSS/MnO_2_ NP electrodes decrease and increase with deposition time, respectively, presenting a trade-off between the two. Therefore, by controlling the spin-coating speed, we prepared PEDOT:PSS electrodes with different transparencies to compare with PEDOT:PSS/MnO_2_ NP electrodes with similar transparency, aiming to ascertain the optimal parameter of the deposition time. The relationship between the areal capacitance and transparency of PEDOT:PSS electrodes and PEDOT:PSS/MnO_2_ NP electrodes with various transparencies is shown in Figure 6d. Within the range of tested transparencies, the areal capacitance of PEDOT:PSS/MnO_2_ NP electrodes is consistently higher than that of PEDOT:PSS electrodes. This outstanding electrochemical property is due to the full integration of the advantages of PEDOT:PSS and MnO_2_ in PEDOT:PSS/MnO_2_ NP electrodes, which serves as transparent current collector and provides high pseudocapacitance. The improvement rate increases first and then descends with the decrease in transparency (i.e., extension of deposition time), rising from 65.6% at a transparency of 83.5% (deposition time of 10 s) to 93.1% at a transparency of 81.3% (deposition time of 40 s), and then decreasing to 80.3% at a transparency of 79.4% (deposition time of 60 s). A similar trend is also observed with 5 mV s^−1^ and 500 mV s^−1^ (Figure S1). Therefore, 40 s is an ideal deposition time parameter, and electrodes prepared via this parameter were used for assembling the supercapacitor.

### 3.3. Characterization of PEDOT:PSS/MnO_2_ NP Supercapacitor

**Figure 7:**
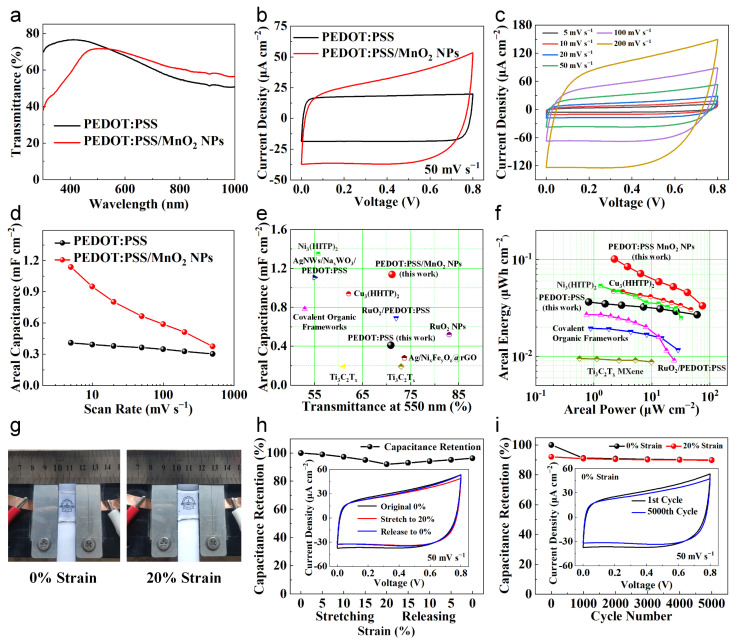
Properties of PEDOT:PSS and PEDOT:PSS/MnO_2_ NPs transparent and stretchable supercapacitors. (**a**) Transmittance spectra of these two supercapacitors. (**b**) CV curves of these supercapacitors with a sweep rate of 50 mV s^−1^. (**c**) CV curves of the PEDOT:PSS/MnO_2_ NP supercapacitor. (**d**) Comparison of areal capacitance of these supercapacitors at various scan rates. (**e**) Comparison of areal capacitance and transparency of these two supercapacitors with other transparent supercapacitors [34,35,42,43,44,45,46,47,48]. (**f**) Ragone plot of these two supercapacitors compared with other transparent symmetric supercapacitors [34,43,44,45,47]. (**g**) Photos of the PEDOT:PSS/MnO_2_ NP supercapacitor under different strains. (**h**) Capacitance retention of the PEDOT:PSS/MnO_2_ NP supercapacitor during the stretching and releasing process with different strains. The CV curves are shown in the inset. (**i**) Capacitance maintenance of the PEDOT:PSS/MnO_2_ NP supercapacitor during 5000 cycles of 20% strain, stretching and releasing. CV curves of the 1st and 5000th cycles are shown in the inset.

To evaluate the performance of PEDOT:PSS/MnO_2_ NPs at the device level, we assembled an all-solid-state, stretchable and transparent supercapacitor using PVA/LiCl gel as the electrolyte. As a comparison, we also assembled a PEDOT:PSS-based supercapacitor with similar transparency. The transparency spectra of two devices are shown in Figure 7a, with transparencies of 70.9% and 71.2% at 550 nm, respectively. The electrochemical property of these supercapacitors was evaluated at room temperature using a two-electrode setup. Figure 7b shows the typical CV curves with a sweep rate of 50 mV s^−1^. Both CV curves are nearly rectangular, and the area enclosed by the PEDOT:PSS/MnO_2_ NP supercapacitor curve is significantly larger than the PEDOT:PSS supercapacitor.

The CV curves and corresponding areal capacitance of the PEDOT:PSS/MnO_2_ NP supercapacitor at different sweep rates are demonstrated in Figure 7c and Figure 7d, respectively. The CV curves remain approximately rectangular even at scan rates up to 200 mV s^−1^. Its areal capacitance at 5 mV s^−1^, 50 mV s^−1^ and 500 mV s^−1^ are 1.14 mF cm^−2^, 0.66 mF cm^−2^ and 0.37 mF cm^−2^, respectively, which are 178%, 83% and 24% higher than that of the PEDOT:PSS supercapacitor. This is because the addition of MnO_2_ NPs increases the capacitance but also increases the electrode resistance, reducing the capacitance enhancement at high scan rates. Additionally, we performed electrochemical impedance spectroscopy measurements in the frequency range of 1 MHz to 0.1 Hz with a signal amplitude of 5 mV. As shown in Figure S2, the semicircle in the high-frequency region of the Nyquist plot is negligible, indicating that the charge transfer resistance (Rct) at the electrode and electrolyte interface is very small. The slope in the low-frequency region further confirms its ideal capacitive behavior. From the Bode plot (Figure S3), the phase angle tail is about −50.2°, which highlights the pseudo-capacitive nature of the PEDOT:PSS/MnO_2_ NP supercapacitor.

We compared the transmittance and areal capacitance of the supercapacitors in this study with advanced transparent devices reported in other work [34,35,42,43,44,45,46,47,48], as shown in Figure 7e. The transparency and areal capacitance of the PEDOT:PSS/MnO_2_ NP supercapacitor are higher than those of most other devices. Its areal energy and areal power were further calculated and compared with other transparent devices (Figure 7f). The symmetric PEDOT:PSS/MnO_2_ NP supercapacitor has a highest areal energy of 0.10 μWh cm^−2^ at an areal power of 2.28 μW cm^−2^, which is outperformed to other transparent symmetric devices based on Ni_3_(HITP)_2_ metal–organic frameworks [47], Cu_3_(HHTP)_2_ metal–organic frameworks [43], RuO_2_/PEDOT:PSS [34], covalent organic frameworks [45], and Ti_3_C_2_T_x_ MXene [44] (Table S1).

To investigate the feasibility of applying PEDOT:PSS/MnO_2_ NP supercapacitor in wearable devices, we evaluated the electrochemical performance of the device under mechanical deformation. We fixed the supercapacitor on a homemade motorized stretching device (Figure S4), and cyclically stretched and released it at a speed of 1 mm s^−1^, with a stretching strain of 20%. Figure 7g shows the photos of the supercapacitor fixed on the device under different strains. It can be seen that the device is highly transparent and shows no obvious damage in the deformed state. During the first stretch–release cycle, we tested the CV curve and calculated the remaining capacitance of the device at every 5% strain change (Figure 7h). When the supercapacitor was stretched from its original state to 20% strain, the CV curve showed slight contraction deformation, and the capacitance gradually decreased to 92.06% of the initial value. The performance degradation of the supercapacitor during the stretching process may primarily be due to the contact degradation between the electrode and the gel electrolyte during deformation, which leads to an increase in the overall resistance of the supercapacitor [49]. When released back to 0% strain, the CV curve partially recovered its shape, and the capacitance gradually increased to 96.28% of the initial value.

The stretching stability of the PEDOT:PSS/MnO_2_ NPs device was further analyzed by subjecting the device to 5000 cycles of 20% strain stretching and releasing. Every 1000 cycles, we tested the CV curves of the supercapacitor at 0% and 20% strain and calculated the capacitance retention (Figure 7i). Even after 5000 cycles, the device retained 89.92% of its original capacity, demonstrating good stretch stability. Most of the performance degradation occurred during the first 1000 cycles (9.10% loss), after which the performance remained nearly stable, and the performance was consistent at 0% and 20% strain. The exceptional stretchability and stretching stability are attributed to the dispersed distribution of MnO_2_ NPs, which helps maintain the mechanical properties of PEDOT:PSS. 

Table 1 provides a detailed comparison of various transparent supercapacitor devices [34,35,42,43,44,45,46,47,48]. The PEDOT:PSS/MnO_2_ NP based supercapacitor presented in this work demonstrates significantly superior overall performance compared to previously reported devices. In addition to higher transmittance and areal capacitance, it also exhibits stable stretchability. Moreover, the PEDOT:PSS/MnO_2_ NPs discussed in this study show great potential for practical commercial applications. They offer the advantage of low cost since the raw material is inexpensive, and the deposition process occurs at room temperature without the need for elevated temperatures or pressures, thus eliminating the need for complex equipment. Furthermore, the solution-processible synthesis is easily scalable for large-scale production, further reducing costs. Additionally, the deposition process is highly efficient, completing in just 40 s, which is several orders of magnitude faster than most existing studies. These exceptional advantages demonstrate the great potential of PEDOT:PSS/MnO_2_ NPs in fabricating transparent and stretchable supercapacitors for powering wearable electronics.

## 4. Conclusions

This study successfully developed a high-performance, stretchable, and transparent supercapacitor by combining PEDOT:PSS with MnO_2_ nanoparticles. The preparation process involved spin-coating PEDOT:PSS films onto a PDMS substrate, followed by electrochemical deposition of MnO_2_ nanoparticles. This method ensured that the nanosized MnO_2_ particles were uniformly distributed, maintaining the transparency and stretchability of PEDOT:PSS. The assembled symmetric supercapacitor demonstrated an outstanding areal capacitance of 1.14 mF cm^−2^ with a transparency of 71.2%, and retained 89.92% of its initial capacitance after 5000 cycles of 20% strain. The use of earth-abundant MnO_2_ and a scalable, solution-processable method offers a cost-effective approach to fabricating supercapacitors. These findings demonstrate the feasibility of producing supercapacitors that combine high capacity, transparency, and mechanical stretchability, making them suitable for applications in wearable electronics.

## Figures and Tables

**Table 1:** Detailed comparison of PEDOT:PSS/MnO_2_ NP supercapacitor with other transparent supercapacitors.

Electrode Material	Transmittance at 550 nm	Areal Capacitance (mF cm^−2^)	Mechanical Performance	Synthesis Method	Synthesis Condition	Ref.
PEDOT:PSS/ MnO_2_ NPs	71.2% vs. air	1.14	Stretchable, 20% strain	Electrochemical deposition method	Ambient conditions 40 s	This work
AgNWs/Na_x_WO_3_ /PEDOT:PSS	55% vs. air	1.107	Just flexible	Hydro-thermal method	180 °C 20 h	[35]
RuO_2_/ PEDOT:PSS	78% vs. PET (~70% vs. air)	0.69	Just flexible	Hydro-thermal method	180 °C 6 h	[34]
Ni_3_(HITP)_2_	61% vs. air	1.35	Just flexible	Modified air/liquid interfacial method	60 °C 30 min	[47]
Cu_3_(HHTP)_2_	62.1% vs. air	0.939	Just flexible	Layer-by-layer assembly method	Ambient conditions 75 s	[43]
Covalent organic frameworks	53% vs. air	0.784	Just flexible	Nucleophilic aromatic substitution reactions	120 °C 72 h	[45]
Ti_3_C_2_T_x_	61% vs. glass (~55% vs. air)	0.189	Just rigid	Modified minimally intensive layer delamination method	48 h	[42]
RuO_2_ NPs	92.3% vs. ITO/glass (~83% vs. air)	0.52	Just rigid	Phase-transfer and precipitation technique	Room temperature 15 h, then annealing at 200 °C	[46]
Ag/Ni_x_Fe_y_O_z_@rGO	73.7% vs. air	0.282	Just flexible	Microwave-assisted method	160 °C 30 min	[48]
Ti_3_C_2_T_x_	73%	0.192	Just flexible	Pressure-less sintering method	30 MPa 1350 °C 1 h	[44]

## Data Availability

The original contributions presented in the study are included in the article and Supplementary Materials; further inquiries can be directed to the corresponding authors.

## Supplementary Materials

The following supporting information can be downloaded at: https://www.mdpi.com/article/10.3390/nano14131080/s1, Table S1: The number and average diameter of MnO_2_ nanoparticles at different deposition times in Figure 2. Figure S1: Areal capacitance versus electrode transparency at 550 nm. (a) Scan rate = 5 mV s^−1^. (b) Scan rate = 500 mV s^−1^. Figure S2: Nyquist plot of the PEDOT:PSS/MnO_2_ NP supercapacitor. Figure S3: Bode plot of the PEDOT:PSS/MnO_2_ NPs supercapacitor. Figure S4: Digital photograph of the homemade motorized stretching device. Table S2: Data of the Ragone plot in Figure 7f. Refs. [34,42,43,45,47] are cited in the Supplementary Materials.

## References

[1] Ray T.R., Choi J., Bandodkar A.J., Krishnan S., Gutruf P., Tian L., Ghaffari R., Rogers J.A. (2019). Bio-Integrated Wearable Systems: A Comprehensive Review. Chem. Rev..

[2] Ates H.C., Nguyen P.Q., Gonzalez-Macia L., Morales-Narváez E., Güder F., Collins J.J., Dincer C. (2022). End-to-end design of wearable sensors. Nat. Rev. Mater..

[3] Pyun K.R., Rogers J.A., Ko S.H. (2022). Materials and devices for immersive virtual reality. Nat. Rev. Mater..

[4] Qi D., Zhang K., Tian G., Jiang B., Huang Y. (2021). Stretchable Electronics Based on PDMS Substrates. Adv. Mater..

[5] Zhao Z., Xia K., Hou Y., Zhang Q., Ye Z., Lu J. (2021). Designing flexible, smart and self-sustainable supercapacitors for portable/wearable electronics: From conductive polymers. Chem. Soc. Rev..

[6] Ye Y., Jiang F. (2022). Highly stretchable, durable, and transient conductive hydrogel for multi-functional sensor and signal transmission applications. Nano Energy.

[7] Won D., Bang J., Choi S.H., Pyun K.R., Jeong S., Lee Y., Ko S.H. (2023). Transparent Electronics for Wearable Electronics Application. Chem. Rev..

[8] Chen L., Khan A., Dai S., Bermak A., Li W.-D. (2023). Metallic Micro-Nano Network-Based Soft Transparent Electrodes: Materials, Processes, and Applications. Adv. Sci..

[9] Chang X., Chen L., Chen J., Zhu Y., Guo Z. (2021). Advances in transparent and stretchable strain sensors. Adv. Compos. Hybrid Mater..

[10] Lim Y.-W., Jin J., Bae B.-S. (2020). Optically Transparent Multiscale Composite Films for Flexible and Wearable Electronics. Adv. Mater..

[11] Zhang L., Jia K., Wang J., Zhao J., Tang J., Hu J. (2022). Stretchable and transparent ionogel-based heaters. Mater. Horiz..

[12] Guo X., Yang F., Sun X., Bai Y., Liu G., Liu W., Wang R., He X. (2022). Anti-Freezing Self-Adhesive Self-Healing Degradable Touch Panel with Ultra-Stretchable Performance Based on Transparent Triboelectric Nanogenerators. Adv. Funct. Mater..

[13] Huang J., Lu Z., He J., Hu H., Liang Q., Liu K., Ren Z., Zhang Y., Yu H., Zheng Z. (2023). Intrinsically stretchable, semi-transparent organic photovoltaics with high efficiency and mechanical robustness via a full-solution process. Energy Environ. Sci..

[14] Debe M.K. (2012). Electrocatalyst approaches and challenges for automotive fuel cells. Nature.

[15] Kodama K., Nagai T., Kuwaki A., Jinnouchi R., Morimoto Y. (2021). Challenges in applying highly active Pt-based nanostructured catalysts for oxygen reduction reactions to fuel cell vehicles. Nat. Nanotechnol..

[16] Palacín M.R., de Guibert A. (2016). Why do batteries fail?. Science.

[17] Zhong M., Zhang M., Li X. (2022). Carbon nanomaterials and their composites for supercapacitors. Carbon Energy.

[18] Cheng X.-B., Zhang R., Zhao C.-Z., Zhang Q. (2017). Toward Safe Lithium Metal Anode in Rechargeable Batteries: A Review. Chem. Rev..

[19] Huang J., Xie Y., You Y., Yuan J., Xu Q., Xie H., Chen Y. (2023). Rational Design of Electrode Materials for Advanced Supercapacitors: From Lab Research to Commercialization. Adv. Funct. Mater..

[20] Nasrin K., Sudharshan V., Subramani K., Sathish M. (2022). Insights into 2D/2D MXene Heterostructures for Improved Synergy in Structure toward Next-Generation Supercapacitors: A Review. Adv. Funct. Mater..

[21] Panda S., Deshmukh K., Khadheer Pasha S.K., Theerthagiri J., Manickam S., Choi M.Y. (2022). MXene based emerging materials for supercapacitor applications: Recent advances, challenges, and future perspectives. Coord. Chem. Rev..

[22] Lee J.-H., Yang G., Kim C.-H., Mahajan R.L., Lee S.-Y., Park S.-J. (2022). Flexible solid-state hybrid supercapacitors for the internet of everything (IoE). Energy Environ. Sci..

[23] Islam M.R., Afroj S., Novoselov K.S., Karim N. (2022). Smart Electronic Textile-Based Wearable Supercapacitors. Adv. Sci..

[24] Fan X., Nie W., Tsai H., Wang N., Huang H., Cheng Y., Wen R., Ma L., Yan F., Xia Y. (2019). PEDOT:PSS for Flexible and Stretchable Electronics: Modifications, Strategies, and Applications. Adv. Sci..

[25] Gueye M.N., Carella A., Faure-Vincent J., Demadrille R., Simonato J.-P. (2020). Progress in understanding structure and transport properties of PEDOT-based materials: A critical review. Prog. Mater. Sci..

[26] Donahue M.J., Sanchez-Sanchez A., Inal S., Qu J., Owens R.M., Mecerreyes D., Malliaras G.G., Martin D.C. (2020). Tailoring PEDOT properties for applications in bioelectronics. Mater. Sci. Eng. R Rep..

[27] Jiang Y., Liu T., Zhou Y. (2020). Recent Advances of Synthesis, Properties, Film Fabrication Methods, Modifications of Poly(3,4-ethylenedioxythiophene), and Applications in Solution-Processed Photovoltaics. Adv. Funct. Mater..

[28] Volkov A.V., Wijeratne K., Mitraka E., Ail U., Zhao D., Tybrandt K., Andreasen J.W., Berggren M., Crispin X., Zozoulenko I.V. (2017). Understanding the Capacitance of PEDOT:PSS. Adv. Funct. Mater..

[29] Choi C., Ashby D.S., Butts D.M., DeBlock R.H., Wei Q., Lau J., Dunn B. (2020). Achieving high energy density and high power density with pseudocapacitive materials. Nat. Rev. Mater..

[30] Zhang X., Liu X., Zeng Y., Tong Y., Lu X. (2020). Oxygen Defects in Promoting the Electrochemical Performance of Metal Oxides for Supercapacitors: Recent Advances and Challenges. Small Methods.

[31] Yue T., Shen B., Gao P. (2022). Carbon material/MnO2 as conductive skeleton for supercapacitor electrode material: A review. Renew. Sustain. Energy Rev..

[32] Ma Y., Xie X., Yang W., Yu Z., Sun X., Zhang Y., Yang X., Kimura H., Hou C., Guo Z. (2021). Recent advances in transition metal oxides with different dimensions as electrodes for high-performance supercapacitors. Adv. Compos. Hybrid Mater..

[33] Kandasamy M., Sahoo S., Nayak S.K., Chakraborty B., Rout C.S. (2021). Recent advances in engineered metal oxide nanostructures for supercapacitor applications: Experimental and theoretical aspects. J. Mater. Chem. A.

[34] Zhang C., Higgins T.M., Park S.-H., O’Brien S.E., Long D., Coleman J.N., Nicolosi V. (2016). Highly flexible and transparent solid-state supercapacitors based on RuO_2_/PEDOT:PSS conductive ultrathin films. Nano Energy.

[35] Huang W.-M., Hsu C.-Y., Chen D.-H. (2022). Sodium tungsten oxide nanowires-based all-solid-state flexible transparent supercapacitors with solar thermal enhanced performance. Chem. Eng. J..

[36] Cheng T., Zhang Y.-Z., Zhang J.-D., Lai W.-Y., Huang W. (2016). High-performance free-standing PEDOT:PSS electrodes for flexible and transparent all-solid-state supercapacitors. J. Mater. Chem. A.

[37] Savagatrup S., Chan E., Renteria-Garcia S.M., Printz A.D., Zaretski A.V., O’Connor T.F., Rodriquez D., Valle E., Lipomi D.J. (2015). Plasticization of PEDOT:PSS by Common Additives for Mechanically Robust Organic Solar Cells and Wearable Sensors. Adv. Funct. Mater..

[38] Vosgueritchian M., Lipomi D.J., Bao Z. (2012). Highly Conductive and Transparent PEDOT:PSS Films with a Fluorosurfactant for Stretchable and Flexible Transparent Electrodes. Adv. Funct. Mater..

[39] Su Z., Yang C., Xu C., Wu H., Zhang Z., Liu T., Zhang C., Yang Q., Li B., Kang F. (2013). Co-electro-deposition of the MnO2–PEDOT:PSS nanostructured composite for high areal mass, flexible asymmetric supercapacitor devices. J. Mater. Chem. A.

[40] Kiruthika S., Sow C., Kulkarni G.U. (2017). Transparent and Flexible Supercapacitors with Networked Electrodes. Small.

[41] Wei W., Cui X., Chen W., Ivey D.G. (2011). Manganese oxide-based materials as electrochemical supercapacitor electrodes. Chem. Soc. Rev..

[42] Guo T., Zhou D., Deng S., Jafarpour M., Avaro J., Neels A., Heier J., Zhang C. (2023). Rational Design of Ti_3_C_2_T_x_ MXene Inks for Conductive, Transparent Films. ACS Nano.

[43] Zhao W., Chen T., Wang W., Bi S., Jiang M., Zhang K.Y., Liu S., Huang W., Zhao Q. (2021). Layer-by-Layer 2D Ultrathin Conductive Cu_3_(HHTP)_2_ Film for High-Performance Flexible Transparent Supercapacitors. Adv. Mater. Interfaces.

[44] Wen D., Wang X., Liu L., Hu C., Sun C., Wu Y., Zhao Y., Zhang J., Liu X., Ying G. (2021). Inkjet Printing Transparent and Conductive MXene (Ti_3_C_2_T_x_) Films: A Strategy for Flexible Energy Storage Devices. ACS Appl. Mater. Interfaces.

[45] Wang W., Zhao W., Chen T., Bai Y., Xu H., Jiang M., Liu S., Huang W., Zhao Q. (2021). All-in-One Hollow Flower-Like Covalent Organic Frameworks for Flexible Transparent Devices. Adv. Funct. Mater..

[46] Ryu I., Kim D., Choe G., Jin S., Hong D., Yim S. (2021). Monodisperse RuO_2_ nanoparticles for highly transparent and rapidly responsive supercapacitor electrodes. J. Mater. Chem. A.

[47] Zhao W., Chen T., Wang W., Jin B., Peng J., Bi S., Jiang M., Liu S., Zhao Q., Huang W. (2020). Conductive Ni_3_(HITP)_2_ MOFs thin films for flexible transparent supercapacitors with high rate capability. Sci. Bull..

[48] Liu T., Yan R., Huang H., Pan L., Cao X., deMello A., Niederberger M. (2020). A Micromolding Method for Transparent and Flexible Thin-Film Supercapacitors and Hybrid Supercapacitors. Adv. Funct. Mater..

[49] Zhou Y., Maleski K., Anasori B., Thostenson J.O., Pang Y., Feng Y., Zeng K., Parker C.B., Zauscher S., Gogotsi Y. (2020). Ti3C2Tx MXene-Reduced Graphene Oxide Composite Electrodes for Stretchable Supercapacitors. ACS Nano.

