# Supplementary material for: MnO2 Nanoparticles Decorated PEDOT:PSS for High Performance Stretchable and Transparent Supercapacitors
Source: Nanomaterials (Basel). 2024 Jun 24;14(13):1080. doi: 10.3390/nano14131080 (PMC11243227; doi:10.3390/nano14131080)
Supplement: Supplementary file 1 [file nanomaterials-14-01080-s001.zip › nanomaterials-3045161-supplementary.pdf]

Supplementary Materials

MnO_2 Nanoparticles Decorated PEDOT:PSS for High Performance Stretchable and Transparent Supercapacitors

Guiming Liu [1], Zhao Huang [1], Jiujie Xu [1], Tiesong Lin [1] , Bowen Zhang [2,*] and Peng He [1,*]

[1] State Key Laboratory of Precision Welding & Joining of Materials and Structures, Harbin Institute of Technology, Harbin 150001, China; liu.guiming@outlook.com (G.L.); huangzhaohit@outlook.com (Z.H.); 20b909121@stu.hit.edu.cn (J.X.); hitjoining@hit.edu.cn (T.L.)

[2] School of Electrical Engineering, Tiangong University, Tianjin, 300350, China

* Correspondence: bowenzhang@tju.edu.cn (B. Z.); hithepeng@hit.edu.cn (P.H.)

Table S1. The number and average diameter of MnO_2 nanoparticles at different deposition times in Figure 2.

Deposition Time (s)	Number of Particles (per μm²)	Average Particle Diameter (nm)
10	12.80	71.36
30	32.91	74.36
60	58.51	69.81

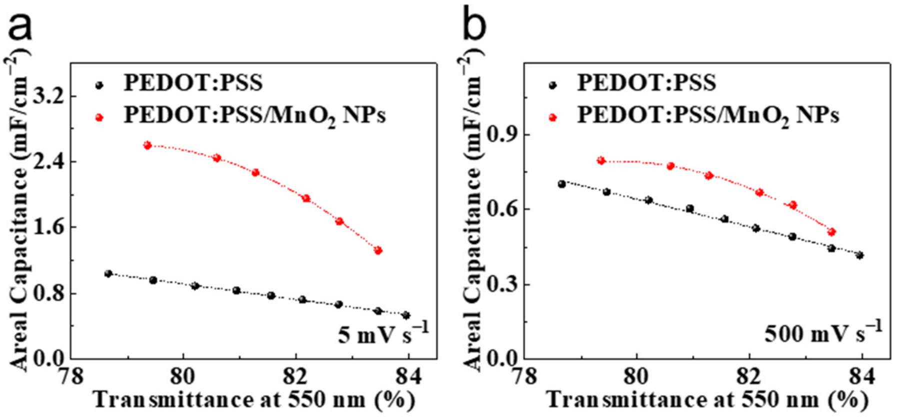

Figure S1. Areal capacity versus electrode transparency at 550 nm. (**a**) Scan rate = 5 mV s^{-1}. (**b**) Scan rate = 500 mV s^{-1}.

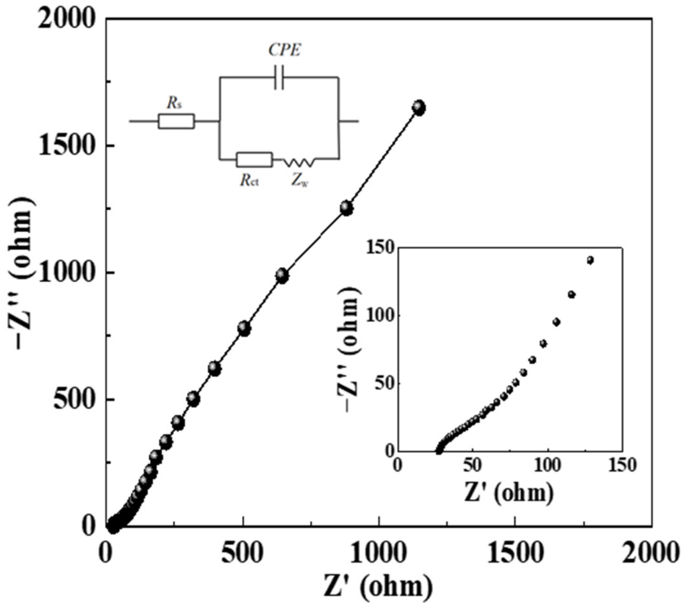

Figure S2. Nyquist plot of the PEDOT:PSS/MnO_2 NPs supercapacitor.

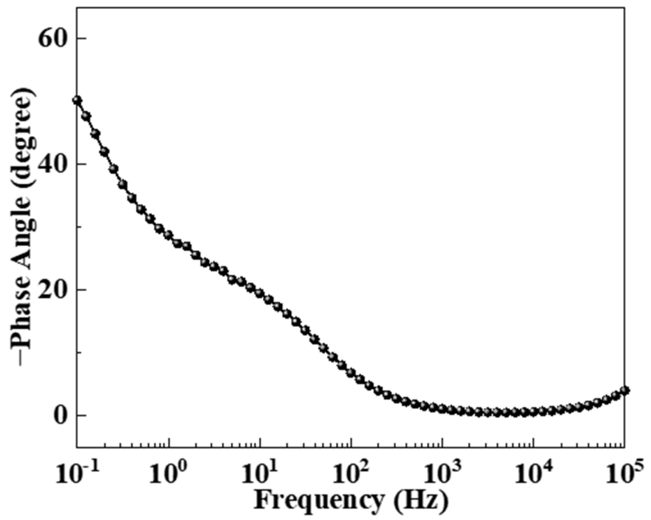

Figure S3. Bode plot of the PEDOT:PSS/MnO_2 NPs supercapacitor.

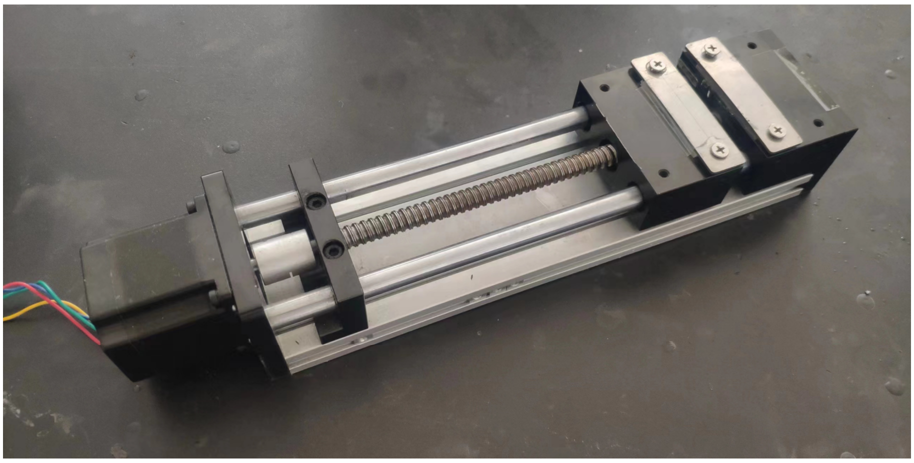

Figure S4. Digital photograph of the homemade motorized stretching device.

Table S2. Data of the Ragone plot in Figure 7f.

Materials	Areal Power ($\mu W\ cm^{-2}$)	Areal Energy ($\mu Wh\ cm^{-2}$)	Ref.
PEDOT:PSS/MnO_2 NPs	2.28	0.101	This work
	3.81	0.085	
	6.43	0.071	
	13.29	0.059	
	23.58	0.05	
	41.00	0.046	
	74.87	0.033	
PEDOT:PSS	0.82	0.036	This work
	1.57	0.035	
	3.03	0.034	
	7.25	0.032	
	13.93	0.031	
	26.21	0.029	
	60.50	0.027	
RuO_2/PEDOT:PSS	0.89	0.0195	[1]
	1.82	0.0192	
	4.35	0.0180	
	8.21	0.0168	
	14.80	0.0155	
	28.32	0.0116	
$Ni_3(HITP)_2$	1.32	0.053	[2]
	2.65	0.047	
	5.22	0.042	
	7.98	0.036	
	13.19	0.035	
	18.79	0.032	
	26.36	0.031	
	31.87	0.025	
$Cu_3(HHTP)_2$	2.20	0.047	[3]
	3.16	0.046	
	6.33	0.043	
	9.50	0.041	
	15.93	0.038	
	22.02	0.036	
	31.57	0.033	
	47.48	0.030	
Covalent Organic Frameworks	0.75	0.027	[4]
	1.32	0.027	
	1.95	0.026	
	2.65	0.025	
	3.96	0.023	
	5.25	0.022	
	7.95	0.020	
	13.13	0.016	
	18.02	0.011	
	24.42	0.009	
$Ti_3C_2T_x$ MXene	0.5682	0.00947	[5]
	1.1244	0.00937	
	2.733	0.00911	
	5.244	0.00874	
	9.792	0.00816	

References

1 Zhang, C.; Higgins, T.M.; Park, S.-H.; O'Brien, S.E.; Long, D.; Coleman, J.N.; Nicolosi, V. Highly flexible and transparent solid-state supercapacitors based on RuO2/PEDOT:PSS conductive ultrathin films. *Nano Energy* **2016**, *28*, 495–505.

2 Zhao, W.; Chen, T.; Wang, W.; Jin, B.; Peng, J.; Bi, S.; Jiang, M.; Liu, S.; Zhao, Q.; Huang, W. Conductive Ni3(HITP)2 MOFs thin films for flexible transparent supercapacitors with high rate capability. *Sci. Bull.* **2020**, *65*, 1803–1811.

3 Zhao, W.; Chen, T.; Wang, W.; Bi, S.; Jiang, M.; Zhang, K.Y.; Liu, S.; Huang, W.; Zhao, Q. Layer-by-Layer 2D Ultrathin Conductive Cu3(HHTP)2 Film for High-Performance Flexible Transparent Supercapacitors. *Adv. Mater. Interfaces* **2021**, *8*, 2100308.

4 Wang, W.; Zhao, W.; Chen, T.; Bai, Y.; Xu, H.; Jiang, M.; Liu, S.; Huang, W.; Zhao, Q. All-in-One Hollow Flower-Like Covalent Organic Frameworks for Flexible Transparent Devices. *Adv. Funct. Mater.* **2021**, *31*, 2010306.

5 Guo, T.; Zhou, D.; Deng, S.; Jafarpour, M.; Avaro, J.; Neels, A.; Heier, J.; Zhang, C. Rational Design of Ti3C2Tx MXene Inks for Conductive, Transparent Films. *ACS Nano* **2023**, *17*, 3737–3749.
